# HETEROGENEITY IN MULTIDIMENSIONAL DEPENDENCY IN OLDER ADULTS IN MEXICO: A LATENT CLASS ANALYSIS APPROACH

**DOI:** 10.1093/geroni/igz038.3052

**Published:** 2019-11-08

**Authors:** Kely Rely, Delfino Vargas-Chanes, Carmen García-Peña, Luis Miguel Gutiérrez-Robledo, Rebeca Wong, Ricardo Ramírez-Aldana

**Affiliations:** 1 National Autonomous University of Mexico, Mexico City, Mexico; 2 Instituto Nacional de Geriatria, Mexico City, Mexico; 3 University of Texas Medical Branch, Galveston, Texas, United States

## Abstract

Background: The number of older adults expected to increase over the coming decades, the public health impact in this population may be substantial, and a greater understanding of the structure underlying risk factor presentation as a potential source of heterogeneity is critical. Objective: Identify and characterize profiles of dependency status in a population of dependent elderly individuals. Methods: The present study is based on the first wave of the Mexican Health Aging Study (MHAS). We included subjects aged 50 or older (n = 13,463 respondents interviewed in 2001). We performed Latent Class Analysis on four domains in older adults’ indicators (physical, psychological, economic and social) to identify distinct classes of dependency profiles. We used LCA to group individuals into homogenous categories of dependency based on observed domains of multidimensional dependency. Multivariable logistic regression was conducted to examine the sociodemographic characteristics associated with each profile. Results: A 4-class solution based on cognitive performance at baseline was the best-fitting model. We characterized the four distinct classes of dependency profiles: active older adult, low, moderate and severe dependency that encompassed multiple dimensions of dependence. Using the “active older adult” class as the reference group, severe dependency, low dependency, and moderate dependency class were more likely to contain females, low education level and poor quality of life 3) the moderate dependency class was less likely to contain cigarette smoking and alcohol user. Conclusions: This study suggests that dependency do not follow a uniform adjustment pattern during the aging process, which reconciles inconsistent previous findings.

